# A New Acquisition and Imaging System for Environmental Measurements: An Experience on the Italian Cultural Heritage

**DOI:** 10.3390/s140509290

**Published:** 2014-05-23

**Authors:** Fabio Leccese, Marco Cagnetti, Andrea Calogero, Daniele Trinca, Stefano di Pasquale, Sabino Giarnetti, Lorenzo Cozzella

**Affiliations:** 1 Science Department, University of “Roma Tre”, Via della Vasca Navale 84, 00146 Rome, Italy; E-Mails: ing.marco.cagnetti@gmail.com (M.C.); andrea.spawn86@gmail.com (A.C.); cico80@gmail.com (D.T.); stefano.dipasquale@uniroma3.it (S.P.); sgiarnetti@uniroma3.it (S.G.); 2 Mathematics and Phisics Department, University of “Roma Tre”, Via della Vasca Navale 84, 00146 Rome, Italy; E-Mail: lorenzo.cozzella@uniroma3.it

**Keywords:** control systems for heritage, remote control for wall painting, monitoring of cultural heritage, wireless networks, ZigBee, ARM processor, artworks preservation

## Abstract

A new acquisition system for remote control of wall paintings has been realized and tested in the field. The system measures temperature and atmospheric pressure in an archeological site where a *fresco* has been put under control. The measuring chain has been designed to be used in unfavorable environments where neither electric power nor telecommunication infrastructures are available. The environmental parameters obtained from the local monitoring are then transferred remotely allowing an easier management by experts in the field of conservation of cultural heritage. The local acquisition system uses an electronic card based on microcontrollers and sends the data to a central unit realized with a Raspberry-Pi. The latter manages a high quality camera to pick up pictures of the fresco. Finally, to realize the remote control at a site not reached by internet signals, a WiMAX connection based on different communication technologies such as WiMAX, Ethernet, GPRS and Satellite, has been set up.

## 1. Introduction

Wall Painting is a very diffuse art expression in Italy. Three marvelous, universally known, examples are the “Sistine Chapel” of Michelangelo in Vatican City, the “Last Supper” of Leonardo da Vinci in Milan, and the “Scrovegni Chapel” of Giotto in Padua. Considering the artistic and scientific relevance of these works, the efforts made to their conservation is remarkable. The favorable geographic collocation of these paintings, often placed in big cities, helps the scientists whose monitoring efforts are often almost exclusively dedicated to preserve them.

However, the vastness of the Italian Artistic Heritage extends across both time and geography, having left traces from prehistory to the present day and everywhere on the Peninsula. In Italy there is scarcely a place that does not have precious artistic evidence that deserves special attention and care. Many times the paintings are located in distant and abandoned places, which often are not supported by adequate infrastructure. The impossibility to transport the wall paintings poses serious logistical difficulties, more than with any other artistic expression. Considering the delicate nature of the paintings and their vulnerability to environmental conditions, continuous monitoring is crucial to program a timely conservative intervention. At the moment, for the most part of the artistic heritage, this monitoring is realized *in loco* obliging the researchers to frequent trips. In the absence of more precise information on the paintings' conditions, these trips are obviously poorly planned. In fact, the occurrence of any problems altering the environmental conditions can be sudden and catastrophic for the paintings and a monitoring schedule too dilated in time may not be sufficient to raise awareness about a threat with sufficient time to enable the appropriate protection and preservation actions.

A data collection system able to convey behavior information to a remote site is a valuable tool for researchers whose transfers to the workplace can then be arranged based on needs.

This work shows a measurement platform based on the latest technologies, which allows remotely checking the environmental conditions of sites where frescoes are located.

The article is divided in the following sections: in Section 2, we present a brief description of the state of the art that allows defining the problem. In Section 3, starting from the description of the architecture of the system, materials and methods used to realize the system are described justifying their choice through comparison with other competitive technologies. Before the conclusions, in Section 4, some tests realized in the field and their results will be presented.

## 2. State of the Art

Data Acquisition System (DAS) for the measurement of physical entities are very common and successfully used in many fields [1–8]. Their use is also growing in some less conventional areas [9–12] and they are seeing more and more applications in the preservation of cultural heritage [13,14], principally in the environmental control of museum rooms. On the contrary, due to the lack of reliability [15], the lack of specific devices that allow overcoming the communication network access problems typical of remote sites, the absence of devices that meet the real control researchers' needs and the reduced energy autonomy in absence of electrical network, DAS are rarely used for preservation of cultural heritage applications placed in remote sites. Scientific progress leads to a constant and continuous improvement/renovation that allows overcoming the existing limits [16]. With this aim, many efforts have been made by researchers to explore satisfying solutions, e.g., some studies are addressed to the realization of specific multi-tier architecture, which decouples the measurement of data from both their memorization and presentation to the final users [17]. Usually, these researches use Wireless Sensor Networks (WSNs) as the first level of the architecture. In fact, WSNs provide autonomous and reliable operations, which reduce the overall costs for structural health monitoring to just a few percentage of those of a conventional cabled monitoring system [15]. Data locally stored are then sent to a remote server using a gateway connected to the Internet where, simply using a browser, the end users can access the information. Among these researches, an interesting artistic in-field application for building preservation is presented in [18], where “SMARTBRICKS” are used as platform to connect sensors to the local gateway; in [19] a WSN transmits the data using the Bluetooth technology; in [20] 2.400–2.483 GHz transmitters designed for their special button sensors are used, while in [17,21,22] ZigBee tx/rx devices are used. Other works aim to improve the already optimal performances of the WSNs, e.g., in [23] a new approach to maximize power saving of the singular nodes is developed, while in [24], the use of solar energy to supply the sensors is investigated. Other interesting works are instead focused on the software management of the WSN [25,26].

All these studies have been developed for environments considered non-hostile, where electrical energy and telecommunication facilities are available, in fact they usually use a personal computer as local gateway and, to send local data, they use the Global System for Mobile (GSM) communication protocol as telecommunication infrastructure.

Although partially inspired by the cited works, our system was created to be used in hostile scenarios, without any available communication facility and without power availability, incorporating a power supply coming from alternative energy sources (such as solar energy) and the ability to transfer data through all the possible communication technologies available on the market, such as GPRS, WiMAX and satellite connections.

The system has the very significant benefit of using standard technologies, such as ARDUINO and ZigBee, allowing high reproducibility and, at the same time, it uses innovative technologies like the Raspberry, which reduces the costs compared to a traditional Personal Computer system. The flexibility of these technologies enables a high scalability of the number and type of sensors that can be used simultaneously. The use of an efficient device like the Raspberry grants the ability to take high quality pictures of the monitored work of art and to make them available to researchers. At the same time, the logic managing the system is innovative because, in order to extend the operational time of the entire system, it is aimed at the minimization of energy consumption.

## 3. Materials and Methods

In this section, the materials and methods used to realize our acquisition system will be shown. First of all, the layered architecture which represents the conceptual base of our system will be described, then, following the presented structure, a detailed description of the single layers will be shown. A comparison with other technologies will also be provided to justify the use of a device rather than others.

### 3.1. The Layered Architecture

**Figure 1.**
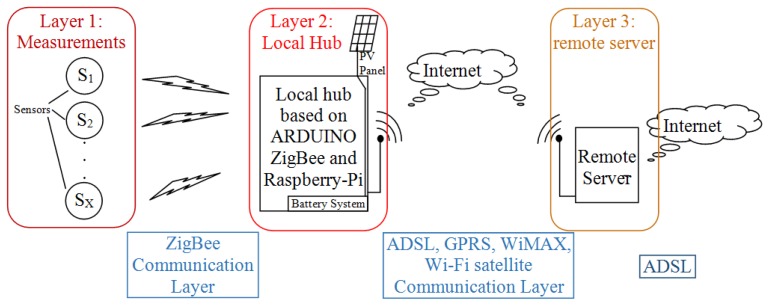
Block Scheme of the measurements system: the layered architecture.

As Figure 1 shows, the whole measurements system has been conceived as a layered structure. This approach allows focusing on the specific functionalities of each single layer. The first layer measures the environmental parameters, the second takes the pictures, and the third shows the collected data. Each layer is autonomous from the others and communicates with the others through an intermediate communication layer.

The first layer is made using electronic control units connected to sensors placed directly in the environment to be monitored. The units are based on ARDUINO technology [27]. The second layer performs the role of a local hub. It collects data from sensor control units and sends them out on demand or at preset times. This is realized by means of a device based on an ARM processor [28–30], in our case a Raspberry-Pi [31–34]. To communicate, the first and the second layer use ZigBee transmitters/receivers. The information gathered by the Raspberry-Pi will be then sent via Internet to a remote server easily accessible by researchers. The hub can be connected to the Internet via any communication protocol whose selection is realizable by software: GPRS, ADSL, Wi-Fi, WiMAX or satellite connection. The third layer is realized by the server, which shows the collected data and pictures. It, by means of a mask, can also communicate with the hub, e.g., asking it to execute some activities rather than automatically. The last layer is the supply one, which provides energy to the hub.

### 3.2. Layer 1: Measurements

The aim of the first layer is to get measurements of environmental entities in the room where the wall painting is placed. The system is realized by some sensors directly connected to an ARDUINO electronic card (also called sensor cards in the remainder of the article). The choice of this card, compared to other possibilities [16], was due to its characteristics which have made it a standard for low frequency control applications. ARDUINO is an open-source electronics prototyping platform based on flexible easy-to-use hardware and software [27]. An ARDUINO board consists of an Atmel 8-bit AVR microcontroller with complementary components to facilitate programming and incorporation into other circuits. An important aspect of the ARDUINO is the standard way that connectors are exposed, allowing the CPU board to be connected to a variety of interchangeable add-on modules known as shields. The ARDUINO board exposes most of the microcontroller's I/O pins for use by other circuits. It provides 20 digital I/O pins, six of which are analog inputs connected to a 10 bits ADC. Several plug-in application shields are also commercially available.

The shield has been designed by us and it is connected to a temperature sensor and a humidity one. The first is MCP9700 with an accuracy of ±4 °C over the 0–70 °C temperature range [35]. The second is a HONEYWELL model HIH-5031-001, with an accuracy of 3% over the 0%–100% humidity range [36]. The need to limit the power consumed obliged us to choose electronics with reduced consumption. The ARDUINO is particularly suited for low energy applications because it is provided with a sleep mode with very low power consumption that is extremely useful when the card is not acquiring data. Other devices, such as data acquisition electronic cards based on Microchip microcontroller [37], Raspberry-Pi [38] or Beaglebone [39] could be used. Table 1 lists some principal characteristics of these devices.

As Table 1 shows, the choice of which device to use to realize the sensor cards depends both on the personal experience of the users in the programming and in the design of electronic cards and on the type of project one wants to develop. If you want to do a software project, the Raspberry-Pi and the Beaglebone are surely the best choice. Their audio, video and internet capabilities make them the winners in this aspect. There is no need to attach external components, so there is no real need to learn electronics and they have the possibility of driving both a GPRS modem and a WiMAX one.

If you want to do a hardware project, the ARDUINO and the Microchip microcontroller family are the best choice. In fact, although the Beaglebone has analog inputs to acquire analog signals, its power consumption is higher than that of the others making it less competitive for extreme applications such as ours, while the Raspberry-Pi has no native analog inputs. The cost is the element that makes ARDUINO the best choice, in fact, although the basic cost promotes the microcontrollers, the need to use external components to work strongly increases the overall cost narrowing the gap. Even more important is the time to develop and test the electronics, equal to one month, while the ARDUINO is immediately ready.

### 3.3. ZigBee Communication Layer

The ZigBee protocol provides an open standard for low-power mesh wireless networking of monitoring and control devices. Working with the IEEE 802.15.4 standard, which focuses on low-rate personal area networking and defines the lower protocol layers (*i.e.*, the physical layer, or PHY, and the medium access control layer, or MAC), ZigBee adds network structure, routing, and security (e.g., key management and authentication) to complete the communications suite. On top of this robust wireless engine, ZigBee profiles provide target applications with the interoperability and intercompatibility required to allow similar products from different manufacturers to work seamless defines the upper layers of the protocol stack, from network to application, including application profiles. ZigBee uses the license-free ISM bands, which provide unrestricted geographic use [40–44].

In the proposed system the ZigBee layer provides the wireless network with the ability to send the environmental parameters, read by the ARDUINO cards, to the local hub or, *vice versa*, to send commands from the hub to the acquisition cards.

The communication network is realized using the XBee radio modules by MaxStream already equipped with an on-chip antenna [45,46]. The characteristics of high radio sensitivity, which reduces the probability to receive corrupted packets (less than 1%), the good operational range (tens of meters indoors) and the low power consumption are particularly suitable for battery-powered applications as ours. In particular, to operate the two-way radio transmission, the necessary current is approximately 50 mA with a 3 V DC source. To insure a further power reduction, the sleep mode ensures a current draw of less than 10 μA.

Nevertheless, the choice to use ZigBee technology is due to its performance. Table 2 shows the performance of ZigBee against those of other two famous technologies used for short-range wireless communications, Bluetooth and Wi-Fi. As the table shows, both Wi-Fi and Bluetooth have higher bit rates than ZigBee, but the higher power consumption suggests that the ZigBee technology is the best candidate to create a sensor network for our application. Furthermore, studies have proved that ZigBee is more affordable in terms of power consumption and costs than competitive technologies [3,29–31].

### 3.4. Measurements Software

To ensure the lowest possible power consumption the firmware, which drives the sensor cards, is conceived to be put the card into sleep mode when it does not need to work. The ARDUINO cards are managed by software written in C that executes the following steps:

(1) wakes up from the sleep mode;
(2) reads data from the sensors;
(3) enables the XBee module;
(4) sends a connection packet toward the hub;
(5) if the hub answers, the ARDUINO sends the data; otherwise waits a connection packet coming from the hub; when it arrives, the ARDUINO sends the message;
(6) disconnects the Xbee module;
(7) goes into sleep mode.

The awakening of the sensor cards is set during the programming phase and managed by the internal watchdog of the ARDUINO. In case of lack of synchronism between the sensor cards and the hub, the cards remain switched on, awaiting the synchronization commands from the hub. The hub, using a specific variable updated in its program, changes the number of cycles of the watchdog of the sensor card to time-align it with the hub.

### 3.5. Layer 2: Local Hub

The aim of layer 2 is to collect data coming from the sensor cards and organize them in ASCII files ready to be read by whatever editor program. This function is generally executed by a Personal Computer (PC) [12,16] which, by means of its operative system, offers all the functionalities needed to collect and show the data. Unluckily the use of PCs often has problems which could be critical in particular applications like ours. For example, the electrical energy consumption could be too high when a battery is used, the reliability of a PC in a hostile environment (too humid and/or too dusty) could be less than what is necessary, and a more reliable PC, such as an industrial one, could be expensive on a limited budget. In order to face these problems, a hub composed by an ARDUINO with a ZigBee tx/rx and a Raspberry-Pi card has been used.

As already shown in Table 1, ARDUINO is advantageous for acquiring data from analog sensors, but it has not all the software features offered by Raspberry-Pi and Beaglebone necessary to create an internet connection. In order to limit the energy needs of the hub, the ARDUINO card plays a fundamental role in the management of the system. In fact, because its low power consumption, it is better to use it as a data collecting card instead of the Raspbery-Pi and to use the latter only for the data transmission to the server. It wakes up at a prefixed time and collects data coming from the sensors card managing all the synchronization tasks for both the sensor cards, and the server. Usually, only one time a day, it, by means of a specific switch, activates the Raspberry-Pi downloading onto it the acquired data. After that, the Raspberry-Pi stores this data and can send it toward the server. If there are particular requests by the server, such as more pictures in a/per day or more environmental measurements, the Raspberry-Pi will change the internal cycle variables to satisfy these exigencies driving also its ARDUINO and the sensor cards.

**Figure 2.**
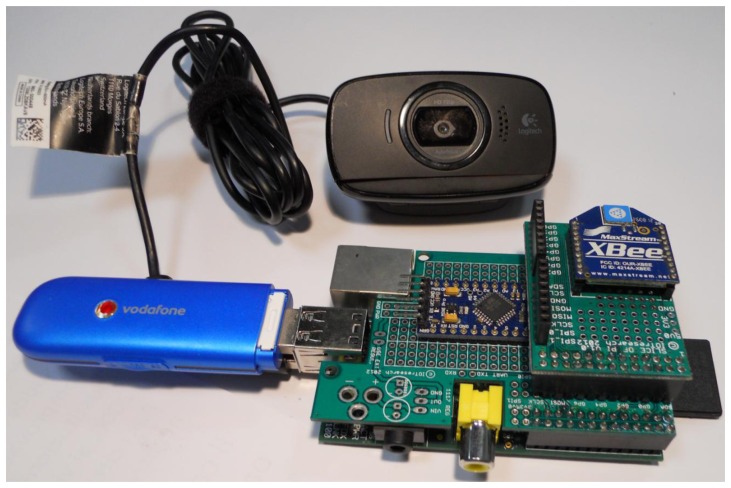
The hub core: Raspberry, ARDUINO with the XBee module and the Camera.

The Raspberry-Pi is a small, barebones computer conceived with the intention of providing low-cost computers and free open-source software. It is a credit card-sized computer powered by the Broadcom BCM2835 system-on-a-chip (SoC). This SoC includes a 32-bit ARM1176JZFS processor, clocked at 700 MHz, and a Videocore IV GPU. It also has 256 MB of RAM in a POP package above the SoC. The Raspberry-Pi is powered by a 5 V micro USB AC charger. While the ARM CPU delivers real-world performance similar to that of a 300 MHz Pentium 2, the Broadcom GPU is a graphics core capable of hardware decoding several high definition video formats. In that regard, the Videocore IV GPU is rather potent as it is capable of hardware decoding 1080p30 H.264 with bit-rates up to 40 Mb/s. The Raspberry-Pi features HDMI and composite video outputs, two USB 2.0 ports, a 10/100 Ethernet port, SD card slot, General Purpose I/O Expansion Board (GPIO) connector, and analog audio output. It only costs 25$ [31–34]. The ARDUINO card is connected to the Raspberry-Pi by the serial port and sends information following the standard serial protocol. Figure 2 shows an image of the naked (without its box) hub.

Connected to the hub there is also a high resolution camera directly driven by the Raspberry-Pi. The camera is a Logitech HD Webcam C525 with a resolution of 8 Megapixels [47]. To obtain the best view, an external 12 V LED flash light driven by the hub software accompanies the webcam. The flash is always connected to the hub and supplied by the battery. Normally the flash is disconnected by the battery to avoid energy loss. When the researchers request a picture, the hub drives the supply circuit that gives energy to the LEDs. The flash is switched on for two seconds before the picture is taken. Obviously, the use of flash must be authorized in order not to cause damage to the painting. The pictures are collected by the Raspberry-Pi and sent along with the environmental data toward the server where it will be then available for further analysis by the researchers.

### 3.6. Hub Software

**Figure 3.**
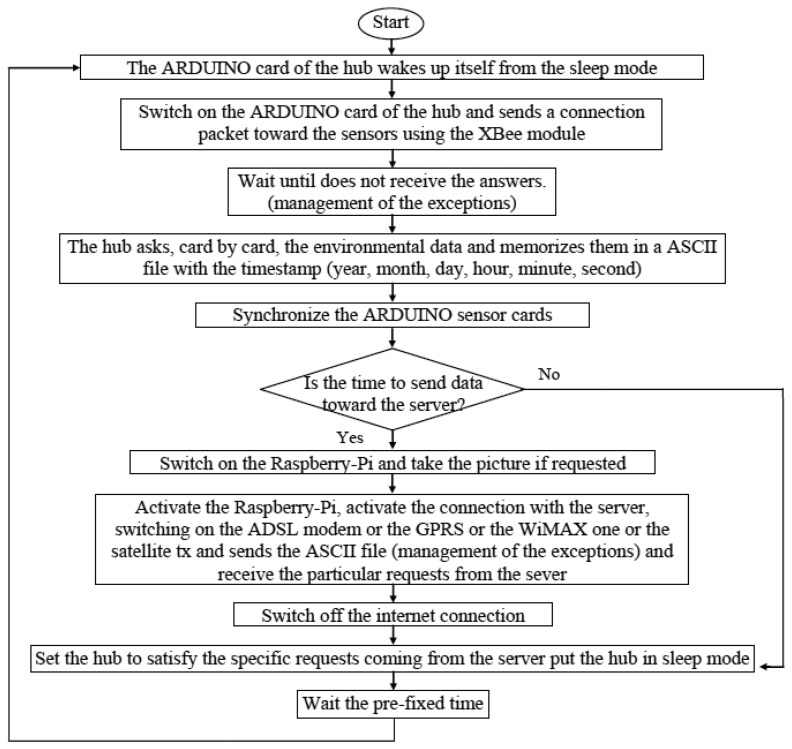
Flow chart of the hub software.

The software on the hub is developed in C Language. It allows to the hub to collect data coming from the ARDUINO cards, to collect pictures of the painting, to also drive the lamp, and, finally, to send the collected information on the Internet toward a server always seeking the greatest energy savings. The software steps are the following (Figure 3).

The algorithm also manages the exceptions. There are two kinds of exceptions: an interruption of the information flux between the sensor cards and the hub, and between the hub and the server. The first can be caused by breakage of the sensor cards, or by the lack of synchronization between these and the hub. In both cases the ARDUINO of the hub is on for 24 h trying to call the apparently broken sensor cards, if they answer, the synchronization procedure is activated; otherwise the hub considers the ARDUINO card broken and an alarm signal is memorized and sent to the server.

If the problem is between the hub and the server, it could be due to the breakage of the server or of the hub, or a communication problem. In the last two cases, the server registers the absence of new data when requested. It repeats the request for one day and, if there is no an answer, an alarm message is activated and sent, using a web service, to the e-mail address(es) of the researchers. In case of the breakage of the server, only a check made by the researchers can solve the problem. In this case, the Raspberry-Pi tries to establish a connection with the server for three hours, after that, using the GPRS modem, it sends an SMS alarm to the mobile phone(s) of the researchers.

### 3.7. Communication Layer

Being the Raspberry-Pi a powerful little computer, it could be used also as a server. This would imply a constant activation of the hub with unavoidable loss of energy during the idle time, a particularly negative condition when there are no mains. To avoid wasting energy, the system has been conceived to be turned on only during the short period of sensor data collection. In this way the data could be viewed only in a very little time window making them *de facto* inaccessible. A remote server allows overcoming this issue. Supposing that the mains are always available, it is always switched on and can always receive data coming from the local hub when it sends them.

To communicate with the server, the local hub can exploit different communication protocols, wired, like ADSL, or wireless as Wi-Fi, GPRS, WiMAX or satellite connections. The first was not taken into account in our application because it is imaginable that if in a place there is an Ethernet jack mains are also present so the place cannot be considered as hostile. The same considerations can be made for Wi-Fi. In fact, Wi-Fi equipment has a limited transmission range, this implies that a Wi-Fi repeater must be placed near the transmitter and always connected to the mains and to an ADSL line. This favorable scenario was not expected in our case. Table 3 shows a synthetic performance comparison between these technologies, while in the next subsections these technologies are better described.

#### 3.7.1. GPRS

General Packet Radio Service (GPRS) is a very widely deployed wireless data service, available with most GSM networks. It offers throughput rates of up to 114 kbps, enabling mobile handsets to access online services at a similar speed to a dial-up modem, but with the convenience of being able to connect from almost anywhere. GPRS enables people to enjoy advanced, feature-rich data services, such as e-mail on the move, multimedia messages, social networking and location-based services.

The RaspBerry-Pi is connected to a GPRS modem, which allows establishing a communication with the remote server. When the software sends an instruction to send data, the Raspberry-Pi switches on the modem and send the files. The modem used is the Huawei E220 USB dongle modem [48]. A similar modem is also connected to the server in such a way to receive the data coming from the hub.

#### 3.7.2. WiMAX

Despite the diffusion of the GSM/GPRS systems, it is not always present in the areas where the sites may be located. In fact, in rural areas, such as ours, a base station for GPRS communication may not be economical in case of low traffic, making the service not profitable for a provider. Usually, the distance covered by a base station is about a hundred meters and in any case not more than a few kilometers [49].

Worldwide Interoperability for Microwave Access (WiMAX) technology is a wireless solution based on the IEEE 802.16 standard which allows to overcome this problem reaching distances of tens of kilometers [50,51] and providing Internet service also in rural areas. By splitting the bandwidth into multiple narrowband channels, WiMAX allows data transport over multiple broad frequency ranges achieving high data rates due to the use of orthogonal frequency division multiplexing (OFDM) [52].

Our system uses a base station (BS) able to spread a WiMAX single carrier TDD service at 3.4845 GHz center frequency and a bandwidth of 10 MHz. As transmitting antenna, the BS uses an Argus model SPPX310M (65 deg Horizontal cut, 6.5 deg Vertical cut) [53] for a total transmitting power of 55 dBm EIRP. The antenna was placed outside on the roof of the church. To communicate between the heritage site and the BS, a Huawei branded HES-319M outdoor WiMAX Customer-Premises Equipment (CPE) [54] has been used. To reliably send data at high speed, the CPE is equipped with a 3.5 GHz 45° Cross-Polarization built-in directional antenna with a maximum power at antenna port of 26 dBm and 14 dBi antenna gain. Furthermore, it avoids any type of interference with the ZigBee standard due to its different operating frequency. The WiMAX module uses 64QAM5/6 modulation for the downlink when the Carrier to Interference-plus-Noise Ratio (CINR) is higher than 30 dB and 16QAM3/4 for the uplink. Instead, when the CINR is lower than 10 dB the link is still possible but using QPSK1/2 modulation because it is more robust at higher noise levels. The CPE is Power over Ethernet (PoE) 802.3 a/f compliant and it runs at 48 VDC, so a DC-DC step-up boost has been required to make the CPE working in our system. We used a LTC3863 [55] mounted on its 1286 A demonstration circuit in order to boost up the 12 V from solar panel to the 48 VDC needed at the CPE PoE connector.

#### 3.7.3. Satellite Connection

Modern satellite Internet service is typically provided to users through geostationary satellites that can offer high data speeds. Satellite Internet generally relies on three components: a satellite in geostationary orbit, a number of ground stations known as “gateways” that relay Internet data to and from the satellite, and a VSAT (very-small-aperture terminal) dish antenna with a transceiver, located at the subscriber's premises and connected to a modem which links the user's network with the transceiver. Satellite Internet services can be provided in two different scenarios: one-way and two-way communications.

One-way communication requires a terrestrial support link for upload (for example the GPRS network); two-way communication is only satellite, suitable for our needs. For our tests, the “Tooway” internet satellite service with the ViaSat RM4100 and a 70 cm diameter dish antenna was chosen; the ViaSat modem provides an Ethernet connection to the Raspberry-Pi, operating at 20 Mbps in downstream and 6 Mbps in upstream mode.

### 3.8. Remote Server

The Remote Server has been conceived as a computer to collect data. It is located in a place equipped with Ethernet, which allows it to be reached from anywhere, and mains, which provide constant electrical energy.

**Figure 4.**
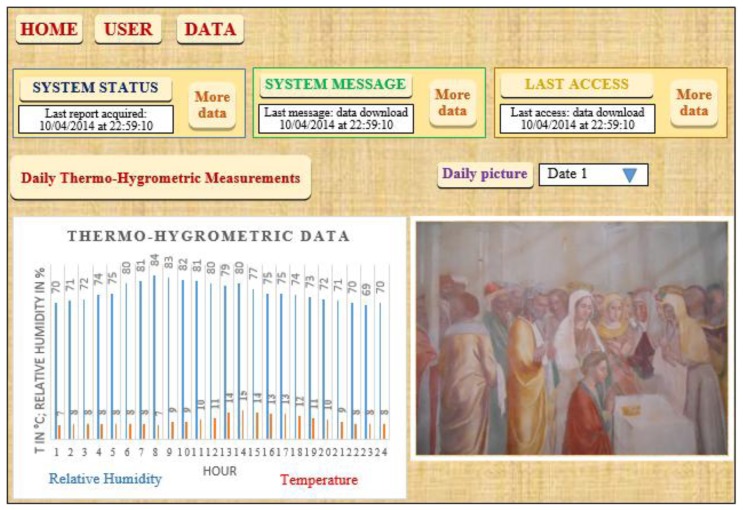
The website.

Being the server a work instrument viewable by authorized experts in the field, following the right path, it is possible to find the pictures and data files inside the right directory. By screening the images, they can decide a prompt on site intervention to protect the painting. On it, a web-oriented software allows authorized researchers to see the data from all over the world. Figure 4 shows the first page of the website where, on the left, one can see the graph with the data coming from the sensors, while, on the right, the frame where the images can be shown is illustrated with an example image. In other secondary pages, it is possible to set the other control parameters such as the number of pictures taken per day, *etc.*

**Figure 5.**
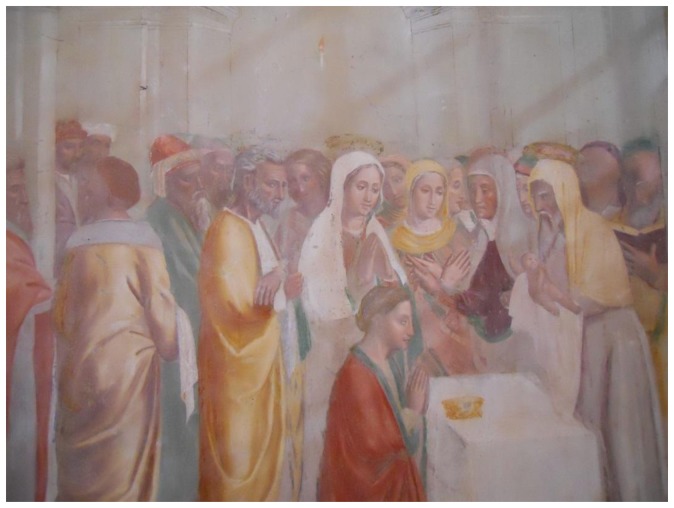
The zoomed image.

Clicking on the picture you can enlarge the image as shown in Figure 5.

### 3.9. Supply Layer

Considering the real possibility of having no mains at the site where the painting is placed, as already explained, the system has been designed to be stand-alone thanks to the energy coming from alternative energy sources. The advantages which may result from these kinds of power supplies are remarkable and well known. In fact, avoiding the expensive wiring of the supply network, there are considerable savings and greater easiness of implementation.

To minimize the battery capacity and the energy required from the solar panel, the circuit has been designed to utilize the lowest power possible. This goal has been achieved through the complex management of the first and second layers. The ARDUINO cards switch themselves on only when the internal watchdog wakes up after a pre-fixed time established during the design phase according to the exigencies of the researchers. In our application, we decided to activate the sensors card every 1 h. During the wake up mode, the cards use about 100 mA, while during the sleep mode the cards absorb 200 μA. The slot time associated to the wake up mode is normally 1 s. This time includes the uncertainty joined with the synchronization with the hub, which, at the same time, should be on and ready to establish the connection with the sensor cards. Considering a normal set-up during the day, the ARDUINO cards are waked up for 24 s while the sleep time is equal to 23 h 59 min and 36 s. This allows an energetic autonomy of the cards of about 1.5 year with four AA batteries of 1.5 V and 2200 mA.

At the same time, the ARDUINO placed on the hub and connected to the Raspberry-Pi, which takes the coordinator role in the ZigBee network, has also the role to activate the hub, opening the supply switch. Even this card is normally in sleep mode. When the watchdog goes up, the ARDUINO wakes up itself and, when prefixed, drives the power electronic switch designed by ourselves that connects the Raspberry-Pi and the battery. At this point, the Raspberry-Pi is supplied and, after the initializing phase, starts the procedure for requesting data from the sensors. Completed this phase, when it is the time to send data toward internet, the Raspberry-Pi drives the ARDUINO asking it to close the power switch, which supplies the GPRS or the WiMAX or the satellite modem. After the initializing time the Raspberry-Pi sends the data through the modem.

Managing in this way the hub, it is in sleep mode for the most part of the day. The ARDUINO absorption has already been described, while for the Raspberry-Pi the consumption in the “on phase” is lower than 500 mA. For the modem consumption, it depends by the type of transmission. For a GPRS modem the consumption is about 300 mA, for a WiMAX system, it is about 35 mA @ 48 V while for the satellite connection it is about 1 A. In our application, they are normally activated once every day to upload the data but the interval is programmable.

Exceptions to a normal set-up are also planned. There are three kind of possible exceptions: on the sensor side, synchronization problem between cards and hub, or on the hub side. The first is caused by a broken sensor card or by the sudden absence of energy from the battery pack. In both cases, the sensor cards do not transmit anything toward the hub, which does not know if the card is broken or if there is a synchronization problem, therefore it activates a 24 h internal timer. During this time, the hub is always on and constantly pings the cards that have not replied. If they answer, the exception is a lack of synchronization and the time alignment procedure is executed. This procedure sets the ARDUINO clock again to avoid misalignment. Instead, if the internal timer reaches 24 h the cards are evidently broken and an alarm procedure is initiated. Moreover, the hub could also have problems. These could be due to the hub or due to the connections to the Internet. In both cases, an alarm procedure is initiated by the server, which immediately shows on the pc software the problem and contemporaneously sends an alarm to the e-mail(s) of the researchers. In this way, they are quickly informed of the status and can decide what to do. In case the problem is the connection and the hub is working correctly, the hub tries to send data with a frequency four times quicker not once a day but more times every day.

Considering the overall consumption, considering the worst case in which the hub could be on for a day, and considering a reasonable autonomy of one week to allow the researchers to prepare the mission and make the necessary repairs, the battery size is at least equal to 10 Ah. It is supplied by a 12 V, 10 W_pp_ PV panel.

The charging process must be conducted according to the battery features (capacity, voltage, chemistry, *etc.*), producing current until the battery has been completely charged, and then replaced by a stand-by current to compensate the battery self-discharge. The chosen battery recharger (CMP12 JUTA of HARBIN Hopeful STAR [56]) provides voltage regulation of battery charging as a function of temperature and has a built-in electronic protection to contrast overloads, short circuits and overvoltages.

## 4. Experimental Set Up, Test and Results

To verify the behavior of the system some tests have been executed both in laboratory and in a real scenario. The latter is the church of “Santa Maria del Soccorso” located in Corchiano about 70 km, from Rome, Italy (latitude 42.345043, longitude 12.345546) where a sixteen-century wall painting is located. The church is viewable on the Internet at the site http://www.prolocorchiano.com/il-nostro-paese/le-chiese/madonna-del-soccorso/, while the picture viewable in Figure 4 is a fresco by the Zuccari brothers placed in a lateral chapel called “Paradise Chapel” (in Italian “Cappella del Paradiso”). Two sensor cards equipped with a temperature sensor and a humidity one make up the first layer as indicated in Section 3. The cards have been programmed to collect data each 1 h. One time every day the Raspberry-Pi section is switched on, it acquires data from its ARDUINO card, and then it opens a connection with the remote server and uploads the data inside. The ancient church is far from any wired telephone lines; luckily, both GPRS and WiMAX signals are present in the area, while we do not use the mains to test the supply section realized by ourselves. The PV panel has been placed on the roof of the church as is the WiMAX antenna.

### 4.1. Layer 1-2 Tests

In order to verify the overall functionality and seek better performance of the ZigBee network some tests have been performed. As said, some tests were performed at the Electric and Electronic Laboratory Measurements of “Roma Tre” University while others were performed in the real scenario for overall two months. In order to validate the choice to use ZigBee technology, the first test aimed to verify the behavior of the ZigBee transmitters/receivers under variable real-life conditions: clear weather, rain, proximity of electrical or electronic devices possibly interfering with the transmission (such as a Wi-Fi Access Points), the second aimed to confirm the reliability of the communication in the real scenario.

The first tests were performed both outdoors, considering one or more obstacles like trees or hills, and indoors, considering one or more walls between the transmitter and the receiver. All tests were carried out using the Standard XBee modules with patch antenna, which is the hardware used in the sensor card, while the X-CTU software, provided by Digi-MaxStream, has been used as test program. 100,000 transmission tests were carried out for each case, using an appropriate adapter to simulate the retransmission. X-CTU tool, using a terminal connected to an XBee module, sends a packet through the network and verifies that the data are correctly returned back from the XBee module which has received the packet. The obtained results, using the minimum transmission power available, are very satisfactory: all packets arrive to their destination and are correctly returned, obtained an average reliability up to 99.95%. Table 4 shows the results of the ZigBee communication reliability tests.

The first test inside the church has been strongly conditioned from the location of the fresco: the hub has been placed at about 3 m in front of the fresco inside the chapel, while the sensor cards were placed very close to the fresco. Considering the short distance in perfect line of sight between the sensor cards and hub, the communication test, always realized by the previously described procedure, gave us 100% of reliability. More interesting were the tests to verify the maximum allowable distance between the hub and the sensor cards considering that, inside the church, there are two rows of five columns each and the maximum usable diagonal distance is approximately 27 m. Even in this case, the transmission tests gave us a correct messages reliability equal to 100%. Putting the hub outside the church, we registered a quickly decrease in the ability to recognize the signal, in fact, just 5 m from the façade of the church (about 33 m the distance between the hub and the sensor cards) the hub is unable to correctly receive the sent data.

Once verified the reliability of the ZigBee communication technology for our aims, we tested the acquisition cards in the field for two months to verify the overall reliability of the Layers 1-2. To accelerate the test phase we stressed the sensor cards fixing the sending time to once every minute. The acquired data were constantly sent to the hub and no strange values were memorized.

Other tests aimed to verify the behavior of the system if some faults were simulated. First of all, we tested the synchronization recovery procedure both changing in the sensor cards the time to send data and changing in the hub the watchdog delay. In both cases, the system synchronizes itself again. Then we simulated a sensor card broken, switching one of them off. Even in this case the system correctly works sending an error message to the server.

Other tests aimed to verify the rightness of the data coming from the sensors, this is the reason why, a PCE-FWS 20 [57,58] Meteorological Station (MS) has been used. Within the uncertainty range of the sensors and of the MS, the measurements realized with our systems were similar to the MS ones.

### 4.2. Layer 2-3 Tests

For the hub, we simulated all the possible errors managed by the software. The breakage of the ARDUINO card, which does not allow the ZigBee communication, quick battery discharge that signals a problem with the PV panel, no connection with the Internet and faults of the Raspberry-Pi. In all cases, the server correctly signals the problem.

Considering the special features of a WiMAX system, even for the previously described system, some reliability test have been performed. Due to the decay of the working distance with introduction of obstacles leading to multi-path fading, the IEEE 802.16.1 standard [58,59] does not provide any range data achievable with WiMAX devices.

Our system works in a rural area some kilometers away from the Base Station essentially in a Line of Sight (LOS) scenario obtained by mounting the receiving antenna on top of an extendable tripod and placing the system on the roof of the church, whilst the BS was on the roof of a building itself [60].

Carrier to Interference plus Noise Ratio (CINR) and Received Signal Strength Indicator (RSSI) are the considered radio parameters, which allow verifying the reliability of a WiMAX connection. The first is a parameter that judges the quality of the WiMAX signal. The higher the value of the CINR, the biggest throughput a link can maintain. The second is a measurement of the total received power of the frame preamble and is used by the subscriber station to determine the received signal level from the BS [61]. The 802.16 standard supports link adaptive modulation and channel coding (AMC) and it adapts to signal degradation by dropping to a lower modulation [62] in order to maintain a constant bit error rate (BER) of 1 × 10^−6^.

The AMC is controlled on the BS side and it applies the Modulation and Coding Schemes (MCS), which defines the most suitable modulation rate for current radio channel, and leads to the highest data rate possible. WiMAX uses mainly quadrature amplitude modulation (QAM) with its more powerful 64-QAM5/6 in downstream mode; it is optional in upstream [59]. Maximum modulation upstream is usually 16-QAM3/4.

**Figure 6.**
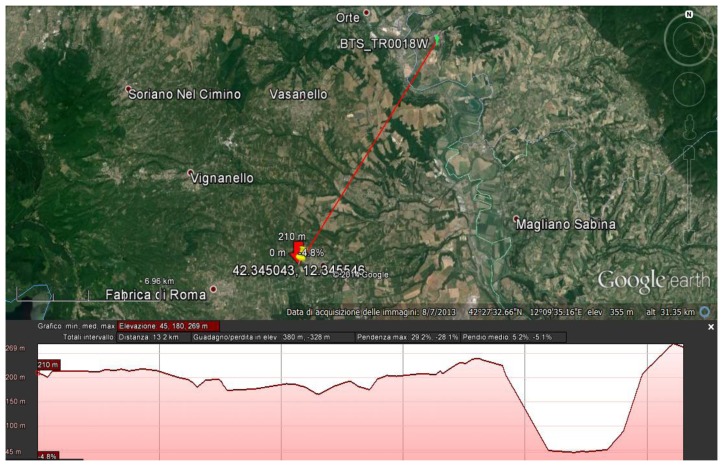
Distance between the site and the BTS with elevation profile.

The nearest BTS antenna offering the WiMAX service is located near Orte, about 13.2 km away from the site of interest. Even if the distance is quite large and beyond the “cell-size” of the BTS (the area covered by the BTS, estimated at about 10 km through simulations), the situation is still lucky since it is almost in LOS (Line Of Sight) as shown in the elevation profile of Figure 6.

Even if the church is placed out of the WiMAX estimated cell edge, the WiMAX radio link has been successfully tested using the outdoors model HES-319M Customer Premise Equipment (CPE) [54]: the links have been correctly established with poor but stable radio parameters that can still guarantee a QPSK1/2 modulation in UpLink and QPSK3/4 in DownLink.

**Figure 7.**
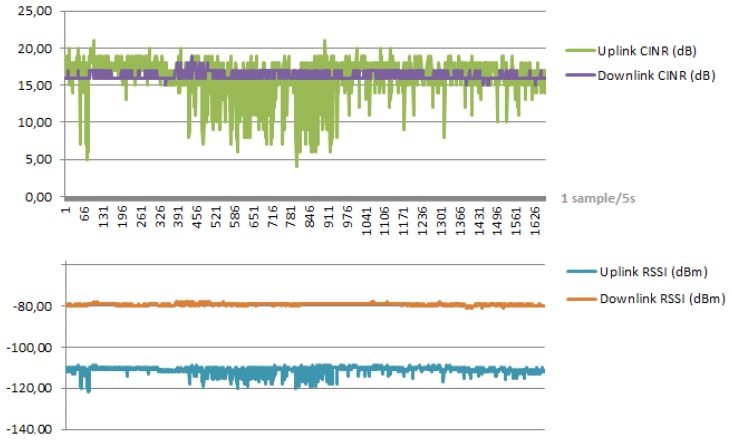
Monitoring of the radio parameters.

As shown in Figure 7, DownLink RSSI at the CPE is about −80 dBm (Received Signal Strength Indication) while UpLink RSSI at the BTS is about −110 dBm. We could not expect higher performances in UpLink, since it is strictly dependent on the maximum output power of the CPE, the carrier operator profiles and the average traffic load on the BTS.

In order to verify the real available bandwidth on the WiMAX connection, the software tool Iperf version 2.0.5 [63,64] has been used to check the TCP throughput; the results are shown in Table 5 where for a single CPE, the optimal number of parallel data streams to obtain the maximum throughput is equal to 5. Default settings of the program fix the duration of the single test reported in the second column, while the third column shows the average bandwidth in that time interval and in this scenario the UpLink throughput is enough to upload a standard 8 MP JPG picture in about 1 min.

## 5. Conclusions

Exploiting new technologies, a new acquisition system for wall painting remote control has been realized and tested in the field. The architecture used provided an affordable and reliable system particularly suitable for hostile environments, which have no wired Internet connections and mains. The system is able to locally check some environmental parameters and send them towards a remote server easily accessible by researchers and experts in the field. Moreover, the system offers the possibility to send high definition pictures, allowing the use of digital zoom to also better view specific particulars of the object under control. The communication layers use the ZigBee technologies for local data transmission and GPRS, WiMAX and satellite technologies for the remote connection.

The energy for the system is provided by an *ad hoc* supply system based on PV panels designed taking into account a series of designing constraints which guarantees the most efficient energy management of the whole system. Test in the field have shown the reliability and the efficacy of the proposed system.

## Figures and Tables

**Table 1.** Arduino *vs.* Microchip microcontrollers, Raspberry-Pi and Beaglebone.

	**Arduino**	**Microchip μC**	**Raspberry-Pi**	**Beaglebone**
**Base price in US $**	35	2÷10	25	50
**External electronic to make an acquisition card for our aim**	$0	$25	$25	$0
**Time required to develop the support external circuitry**	0	1 month	0	1 month
**Power draw**	∼50 mA @ 5 V	∼20 mA @ 5 V	∼150 mA @ 5 V	∼250 mA @ 5 V
**Operating system**	Custom	Custom	Linux	Linux
**Suited for**	Hardware	Hardware	Software	Software
**Number of I/O pins**	14 Digital (6 PWM), 6 analog	5÷28 (Depends by the version); 5÷14 analog	8 Digital, 0 Analog	65 Digital, 7 analog
**Peripherals**	None	None	2 USB hosts, 1 micro-USB power, 1 10/100 Mbps ethernet	1 USB host, 1 mini-USB client, 1 10/100 Mbps ethernet
**Internet**	Via shield	Via shield	Yes	Yes

**Table 2.** ZigBee *vs.* some other wireless network.

**Feature**	**ZigBee**	**Wi-Fi**	**Bluetooth**
IEEE standard	802.15.04	802.11b/g	802.15.01
Main application	Control	Broadband	Mobile devices
Number of network devices	Up to 65,000	32	7
Bit rate	20–250 kb/s	11/54 Mb/s	720 kb/s
Range	100 m	100 m	10 m
Battery life	100–1000 days	1–5 days	1–7 days

**Table 3.** WiMAX *vs.* other communication technologies.

	**WiMAX**	**ADSL**	**Wi-Fi**	**GPRS**	**Satellite Communication**
**Wireless?**	Yes	No	Yes	Yes	Yes

**Distance Covered**	Up to 70 km from the CPE	Everywhere with cable	Up to 100 m	Up to 5 km from the BS	Everywhere the signal satellite is on

**Subscription Costs**	About 20€	About 20 €	About 20€	About 20€	About 100€
**Availability in rural area**	Medium/High	No	No	Medium	Yes

**Table 4.** ZigBee reliability tests.

**XBee Standard—Patch Antenna—Outdoors**				
	Sunny		Rainy	

	50 m	100 m	50 m	100 m

No obstacles	100%	99.99%	99.98%	99.97%
Tree	99.97%	99.96%	99.98%	99.96%

**XBee Standard—Patch Antenna—Indoors—more than 10 m from WiFi AP**				

1 Wall	2 Walls		3 Walls	
100%	99.98%		99.96%	

**XBee Standard—Indoors—5 m from WiFi AP**				

1 Wall	2 Walls		3 Walls	
99.98%	99.88%		99.96%	

**Table 5.** Throughput performance.

**Client Connecting to Iperf Server, TCP port 5001, TCP Window Size: 16.0 Kbyte**			
Direction	No. of parallel streams	Interval (s)	Bandwidth
Uplink	5	17.5	135 Kbps
Downlink	5	13.3	3.56 Mbps
